# High-Quality Draft Genome Sequence of Pseudomonas songnenensis L103, a Denitrifier Isolated from a 100-Meter-Deep Aquifer in a Heavily Nitrogen-Fertilized Agricultural Area

**DOI:** 10.1128/MRA.00373-19

**Published:** 2019-09-05

**Authors:** Mengshuai Liu, Shuaimin Chen, Shiqin Wang, Chunsheng Hu, Binbin Liu

**Affiliations:** a Key Laboratory of Agricultural Water Resources, Hebei Key Laboratory of Soil Ecology, Center for Agricultural Resources Research, Institute of Genetics and Developmental Biology, Chinese Academy of Sciences, Shijiazhuang, China; b University of Chinese Academy of Sciences, Beijing, China; University of Southern California

## Abstract

Pseudomonas songnenensis strain L103 was isolated from a 100-m-deep aquifer in the North China Plain, a heavily nitrogen-fertilized agricultural area. The genome is 4.8 Mb and contains 4,409 protein-coding genes, including a full set of genes (*nar*, *nir*, *nor*, and *nos*) for complete denitrification.

## ANNOUNCEMENT

Pseudomonas songnenensis is a newly described species (1), with strain NEAU-ST5-5 as the type strain, which is isolated from saline and alkaline soils and currently is the only strain published in this species. *Pseudomonas songnenensis* L103 was isolated from a 100-m-deep aquifer sample in a farming area in the Luancheng Argo-ecosystem Experimental Station in the North China Plain (37°53′N, 114°41′E; elevation 50 m). The sampling site had been fertilized with urea at 600 kg N year^−1^ ha^−1^ for ∼20 years (2). The samples for isolating bacteria were collected using a Geoprobe direct-push rig (model 6610DT; Geoprobe Systems, USA). The strain was isolated by serial dilution of soil suspension and forming colonies anaerobically on tryptic soy broth (TSB) medium supplemented with 2 mM KNO_3_ (3).

In order to investigate the genomic characteristics, in particular, the denitrification properties of strain L103, whole-genome sequencing was performed. A single colony was picked from the plate as described above, and the liquid culture in TSB medium was cultivated at 28°C for 12 h. Genomic DNA was extracted using the TIANamp bacterial DNA kit (Tiangen Biotech, Beijing, China) according to the manufacturer’s instructions. The whole-genome shotgun sequencing was performed on the HiSeq platform (Illumina, San Diego, CA, USA) using the paired-end strategy, and the DNA library was constructed using a NEBNext Ultra II DNA library prep kit for Illumina (New England Biolabs, USA). A total of 10,055,782 reads of 150 bp were generated and filtered to remove low-quality reads (quality score, <20) using FASTX-ToolKit version 0.0.13 (4). De novo genome assembly was performed using SPAdes 3.1.1 (5) with the “–careful” option and the k-mer values 21, 33, 55, 67, 77, 89, 99, 101, and 111. The assembly resulted in 18 contigs with 315-fold coverage. The draft genome of strain L103 consists of 4,785,340 bases with an average G+C content of 62.83%. Strain L103 is closely related to type strain NEAU-ST5-5 with an average nucleotide identity (ANI) score (6) of 98.36% and a 16S rRNA gene identity of 99.5%. The functional annotations were performed using the NCBI Prokaryotic Genome Annotation Pipeline. In total, 4,520 genes were predicted, including 4,409 coding sequences (CDS), 61 RNA genes, and 50 pseudogenes. The annotation results indicated that strain L103 harbors a full set of denitrification genes, namely, *nar*, *nir*, *nor*, and *nos*, encoding nitrate reductase, nitrite reductase, nitric oxide reductase, and nitrous oxide reductase, respectively.

### Data availability.

The genome sequence of *P. songnenensis* L103 has been deposited at GenBank under the accession number RWYU00000000. The version described in this paper is the second version, RWYU02000000. The raw sequences are available at the SRA under the accession number SRR8773562.
